# Examination of permittivity for depolarization field of ferroelectric by ab initio calculation, suggesting hidden mechanisms

**DOI:** 10.1038/s41598-021-81237-0

**Published:** 2021-01-25

**Authors:** Yukio Watanabe

**Affiliations:** Kyushu University, Fukuoka, Japan

**Keywords:** Engineering, Materials science, Nanoscience and technology, Physics

## Abstract

Electrostatics of depolarization field *E*_*d*_ in relation to the polarization is studied. In particular, the value of permittivity for *E*_*d*_ (*ε*_*d*_) in prototypical situations of ferroelectrics, including Mehta formula, is examined by ab initio calculations. By using spontaneous polarization *P*_S_ corresponding to accurate experiment ones, we show *ε*_*d*_ = 1, which suggests that the results of *ε*_*d*_ ≫ 1 indicate hidden mechanisms; *ε*_*d*_ = 1 suggests that the effect of *E*_*d*_ is significant to induce intriguing important phenomena overlooked by *ε*_*d*_ ≫ 1. A bridge between *ε*_*d*_ = 1 and *ε*_*d*_ ≫ 1, i.e. the consistency of *ε*_*d*_ = 1 with conventional results is presented. The exact electrostatic equality of head-to-head–tail-to-tail domains to free-standing ferroelectrics is deduced. Hence, most stoichiometric clean freestanding monodomain ferroelectrics and head-to-head–tail-to-tail domains are shown unstable regardless of size, unless partially metallic. This verifies the previous results in a transparent manner*.* This conclusion is shown consistent with a recent hyperferroelectric LiBeSb and “freestanding” monolayer ferroelectrics, of which origin is suggested to be adsorbates. In addition, this restriction is suggested to break in externally strained ultrathin ferroelectrics. The macroscopic formulas of *E*_*d*_ are found valid down to a several unit-cells, when electronic and atomic-scale surface effects are unimportant and accurate *P*_S_ is used.

## Introduction

Ferroelectrics (FEs) have reversible spontaneous polarization *P*_S_ that is useful in various applications, for which high insulativity is desired. Therefore, ideal insulativity of FE is assumed in most studies. In such high insulativity FEs, the depolarization field *E*_*d*_ exists universally even in the absence of external electric field *E*_*ext*_, owing to the charge − *∇*·*P*_S_ originating from inhomogeneity or the existence of surface; For a homogeneous *P*_S_, *E*_*d*_ disappears for infinite FE without surface or FE with no surface effect and ideal metal electrodes.

Although analytical formulas of *E*_*d*_ are unnecessary in ab initio calculations, they are indispensable for non ab initio examinations of *P*_S_ configurations, stability of domains, and critical thicknesses of FEs^1–17^. These formulas use the permittivity for *E*_*d*_ (*ε*_*d*_), of which difference affects critically the results. No controversy exists for *ε*_*d*_ = *ε*_*f*_ used in combination with an initial *P*_S_ that is an ideal bulk *P*_S_ for no macroscopic field in FE as in Kittel models^1,2^. Here, *ε*_*f*_ is static linear permittivity *ε*_*r*_ of FE*.* Otherwise, the choice of *ε*_*d*_ is controversial.

Because the polarization *P* under *E*_*ext*_ is *P* = *P*_S_ + (*ε*_*f*_ − 1)*ε*_0_*E*_*ext*_ (*ε*_0:_ vacuum permittivity) and the permittivity of FE *under* *E*_*d*_ is still *ε*_*f*_ ≫ 1 (“Results”), the standard choice of *ε*_*d*_ is *ε*_*f*_^4–10,16,17^. We define accurate *P*_S_ as *P*_S_ obtained by accurate experiments of ideal samples; Accurate *P*_S_ can be obtained from ion and electron distribution, e.g. by transmission electron microscopy TEM. When a FE is homogeneous or single-domained, accurate *P*_S_ can be obtained by standard electrical measurements (“Results”). An alternate description of accurate *P*_S_ is that *P*_S_ is a total polarization under the influence of *E*_*d*_ and *E*_*ext*_ = 0.

Examples of *ε*_*d*_ = *ε*_*f*_ are followings, which are for single domain homogeneous FEs. In this paragraph, “experimental” *P*_S_ refers to *P*_S_ of each specific sample, which is obtain by the measurements of that sample or is *P*_S_ of similar samples. Mehta et al.^4^ studied *E*_*d*_ in capacitors, using experimental *ε*_*d*_ = *ε*_*f*_ = 1000 and *P*_S_ = 10 μC/cm^2^ obtained from the saturated polarization of thin film capacitors. Black et al.^5^ studied *E*_*d*_ of thin film capacitors, using experimental *ε*_*d*_ = *ε*_*f*_ = 350 obtained from the polarization-hysteresis near saturation and *P*_S_ obtained from the saturated polarization. Zhao et al.^6^ estimated *E*_*d*_ of poly-vinylidenefluoride–trifluoroethylene [P(VDF–TrFE)] thin films by using *E*_*d*_ = − *P*_S_/*ε*_*f*_*ε*_0_ with *P*_S_ from remnant polarization measurements (7 μC/cm^2^) and *ε*_*d*_ = *ε*_*f*_ = 10. In the electrostatic study of Tian et al.^7^, *E*_*d*_ in BiFeO_3_ thin films was estimated by using *ε*_*f*_ = 60 (in Eq. (1) of Ref.^7^), where this *P* is initially a total polarization of a single domain state. Kim et al.^8^ estimated *E*_*d*_ in BaTiO_3_ (BTO) ultrathin films through the formula by Mehta^4^ with the experimental remnant *P* obtained from pulse train methods and *ε*_*d*_ = *ε*_*f*_ = 80 from *ε*_*f*_ − *E*_*ext*_ curves. In the Ginzburg–Landau–Devonshire (GLD) theory of BTO ultrathin films by Jo et al.^9^, *E*_*d*_ was given through the Mehta formula^4^ with the same *P* as *P* in the GLD equation and *ε*_*d*_ = *ε*_*f*_ = 80 of Ref.^8^, where *P* in GLD theory is the total polarization. Schroeder et al.^10^ estimated *E*_*d*_ in HfO_2_ and PZT ultrathin films through the Mehta formula^4^ with experimental *P*_S_ and *ε*_*d*_ = *ε*_*f*_ = 20–300. Similar analyses with *ε*_*d*_ = *ε*_*f*_ ≫ 1 are frequently employed^16,17^.

Contrastingly*,* a primitive considerations show *ε*_*d*_ = 1 for *P*_S_, i.e. a total *P*_S_ (≡ *P*(*E*_*f*_(*E*_*ext*_ = 0)))^13^, where *E*_*f*_ is the total macroscopic electric filed in FE. For freestanding FEs, for example, *E*_*f*_(*E*_*ext*_) = *E*_*ext*_ − *P*(*E*_*f*_(*E*_*ext*_))/*ε*_0_ or *E*_*f*_(*E*_*ext*_) = *E*_*ext*_/*ε*_*f*_ − *P*(*E*_*f*_(*E*_*ext*_ = 0))/*ε*_0_ (“Results”). This implies that *ε*_*d*_ = *ε*_*f*_^4–10,16,17^ may be double counting, while we note that the electrical measurements of *P*_S_^4–10,16,17^ are indirect measurements based on induced charge per area in electrodes *Q*. If *ε*_*d*_ = 1 is correct, the successes of the analyses using *ε*_*d*_ = *ε*_*f*_ are attributed to inappropriate parameters or unidentified screening mechanisms.

We think that the existence of this controversy on *ε*_*d*_ is due to explanations based on macroscopic quantities. Because macroscopic explanations are abstract, they are unsuitable to bridge the gap between two conflicting views of *ε*_*d*_. On the other hand, ab initio estimation of *ε*_*d*_ is considered as the clearest method for this problem but is not reported to our knowledge. Hence, we clarify *ε*_*d*_ in the formulas of *E*_*d*_, by ab initio simulations in which ab initio *P*_S_ is exactly *P*(*E*_*f*_(*E*_*ext*_ = 0)), which is considered as *P*_S_ obtained by accurate experiments of ideal samples. Here, the standard theoretical assumptions: pure, stoichiometric, clean FEs are used.

*E*_*d*_ is related to fundamental issues such as stability of monodomains, critical thicknesses of FE, and the emergence of ferroelectricity in superlattices. Some of these subjects require the consideration of other effects such as strain-induced FE and electronic effect at electrodes^11^. To avoid the complexity, we concentrate on free-standing insulating FE and its electrostatic identicals, i.e. head-to-head–tail-to-tail (HH–TT) domains. Thus, *we estimate the value of ε*_*d*_* in the formula of E*_*d*_ in a clear manner.

As expected from the electronic interaction at the electrode^11^, it may be argued that the formula of *E*_*d*_ based on electrostatics is not possible for nm-FEs. We resolve this by focusing on the formulas of *E*_*d*_ and using ab initio *P*_S_ in the formulas. Therefore, *P*_S_ in these formulas contains the effects of the interactions in slabs or superlattices, whereas the absence of these effects in conventional studies has limited the applicability. The use of nonlinear *ε*_*f*_ is often better than linear *ε*_*f*_ but can be approximated by an average linear *ε*_*f*_^12^. Therefore, the conclusions below are applicable also to the nonlinear *ε*_*f*_ (≫ 1).

## Model

**Figure 1:**
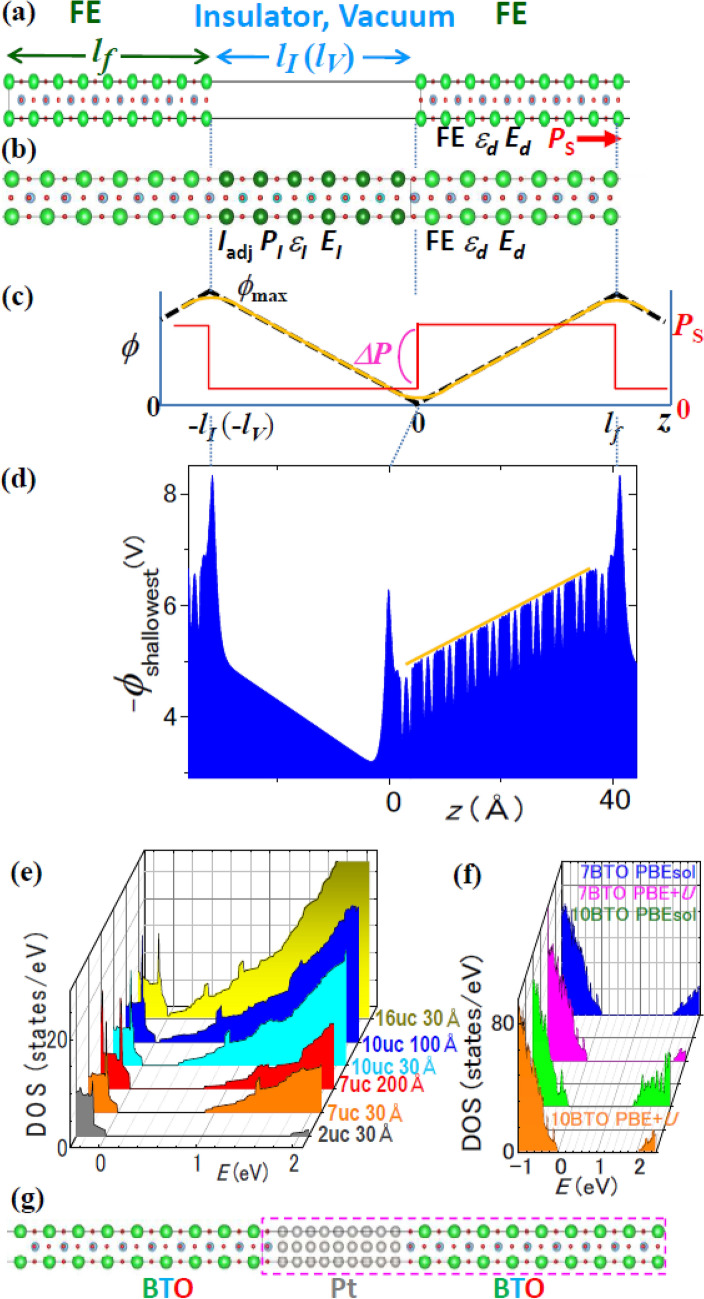
Atomic model of (**a**) FE/vacuum and (**b**) FE/*I*_adj_, where FE is BTO and *I*_adj_ is STO. BTO/STO serves also as a mimic of an inhomogeneous FE. (**c**) Macroscopic model. The minimum and maximum electrostatic potential *ϕ* are 0 and *ϕ*_max_, respectively. (**d**) Example of estimation of *E*_*d*_ from the atomic − *eϕ*. The orange line shows the envelope yielding *E*_*d*_. (**e**) Progressive development of 2D metallic layer: DOS of STO1.005/vacuum, where *l*_*f*_ in unit-cells and *l*_*V*_ in Å are shown on the right. 10-unit-cell-STO with *l*_V_ = 100 Å is marginally insulating, while 16-unit-cell-STO with *l*_V_ = 30 Å is metallic. The insulating slabs are used in Fig. 3. *P*_S_ in the slab was typically 1 μC/cm^2^. (**f**) DOS of 7 and 10-unit-cell BTO/5-unit-cell-STO calculated with PBEsol and PBE + *U*. (**g**) Atomic model of BTO/Pt.

**Figure 2:**
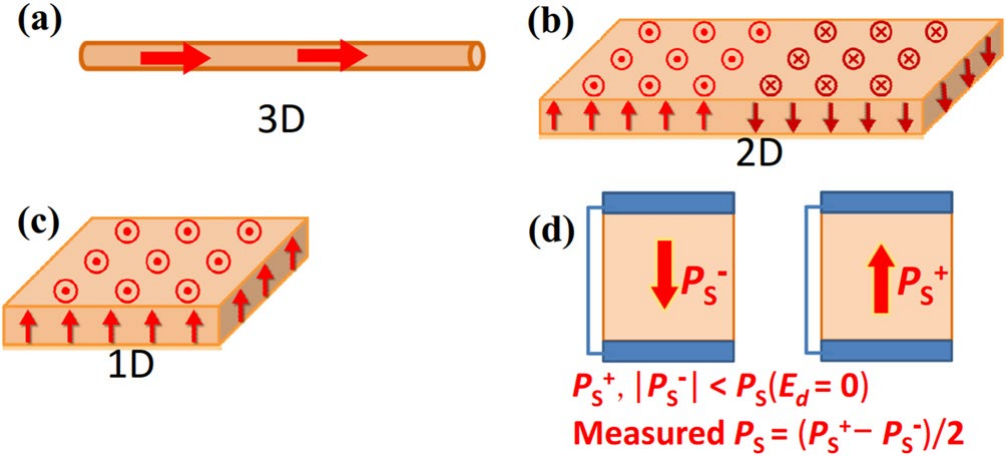
Definition of (**a**) three, (**b**) two, (**c**) one-dimensions for FE in this article. Dimensionality is not referred to the shape of an object. *θ* is the angle between the normal to the surface in (**c**) and the direction of *P*_S_. (**d**) Typical measurement of *P*_S_ in a capacitor. This is smaller than bulk *P*_S_ (*E*_*d*_ = 0), because of nonzero screening length in electrodes.

For simplicity, we discuss 1-dimensional (1D) cases with *E*_*ext*_ = 0, where FEs with thickness *l*_*f*_ are in periodic slabs (Fig. 1). Here, *1D refers not to the shape of object* (Fig. 2) but to the case where all the properties change only along one coordinate; Fig. 1a,b show FE in vacuum with thickness *l*_*V*_ and FE/paraelectric (*I*_adj_) superlattice, respectively, while the latter mimics an inhomogeneous FE. *I*_adj_ stands for both vacuum and insulator, which is dielectric or FE having different *P*_S._ The polarization, thickness, and permittivity of *I*_adj_ are *P*_*I*_, *l*_*I*_, and *ε*_*I*_, respectively, and the thickness of slab is *l*_*SC*_ = *l*_*f*_ + *l*_*I*_ (*l*_*V*_). The angles of the polarization *P* of FE and *I*_adj_ to the slab direction are *θ* and *θ*_*I*_, respectively.

The macroscopic and atomic electrostatic potential (*ϕ*) of these models are represented by Fig. 1c,d, respectively. ab initio *E*_*d*_ (*E*_*d*_^*ab initio*^) was obtained from the envelope of the peak tops of atomic electrostatic potential, of which example is Fig. 1d. All these FE/vacuum and FE/paraelectric exhibited the density of states (DOS) of insulators (Fig. 1e,f). Additionally, BaTiO_3_ (BTO) capacitors are examined, where metal layers are standard electrode materials for FEs: SrRuO_3_ or Pt and ~ 20 Å (Fig. 1g).

Accurate estimation of *P*_S_ is indispensable for estimating *E*_*d*_ correctly and achieved by direct Berry phase calculations. To enable these calculations, we designed special FE slabs and procedures described in “Methods”. This is because stable 1D-FEs in vacuum are metallic^14,15^ and, hence, direct Berry phase calculations are not possible; Even a two unit-cell thick (~ 8 Å) BTO in vacuum is metallic, when FE is enforced^18^. To achieve insulativity, we used tetragonal (P4mm) SrTiO_3_ (STO) of which *a*-axis lattice constant increased by 0.5% and decreased by 0.01% from that of the theoretical cubic phase. For these *a*-axis lattice constants, bulk STO was FE^19^. We refer to them as STO1.005 and STO.9999, respectively, of which bulk *P*_S_’s were 3.56 μC/cm^2^ and 6.15 μC/cm^2^, respectively, by VASP^19–28^.

Macroscopic equations of *E*_*d*_ are obtained in a following manner. The normal component of *P* of FE (*P*_⊥_) under *E*_*d*_ is *P*_⊥_ = *P*_S_ cos *θ* + (*ε*_*d*_ − 1) *ε*_0_*E*_*d*_ in standard approaches^4–10,16,17^. The equation of continuity of electric flux is *P*_S_cos *θ* + *ε*_*d*_*ε*_0_*E*_*d*_ = *P*_*I*_ cos *θ*_*I*_ + *ε*_0_*E*_*I*_, where *E*_*I*_ is the macroscopic electric field in *I*_adj_. The validity of this continuity in the presence of peaks at the surfaces (Fig. 1d) is explained in “Methods”. The continuity of potential in a periodic boundary condition yields *E*_*I*_*l*_*I*_ = − *E*_*d*_*l*_*f*_ (Fig. 1c). Therefore, we have for *θ* = 0

1 $${E}_{d}=-\frac{{P}_{S} - {P}_{I}}{{\varepsilon }_{0}\left({\varepsilon }_{d} +\frac{{l}_{f}}{{l}_{I}}\right)}.$$

For *θ* ≠ 0 and *θ*_*I*_ ≠ 0, Eq. (1) is *E*_*d*_ = − (*P*_S_ cos *θ* − *P*_*I*_ cos *θ*_*I*_)/*ε*_0_(*ε*_*d*_ + *l*_*f*_/*l*_*I*_). In the present study, *P*_S_ and *P*_I_ in Eq. (1) are given by ab initio calculations that simultaneously yield *E*_*d*_ consistent with *P*_S_. Therefore, the only unknown quantity is *ε*_*d*_.

When *P*_I_ = (*ε*_*I*_ − 1)*ε*_0_*E*_*I*_, Eq. (2) for *θ* = 0 is

2 $${E}_{d}=-\frac{{P}_{S}}{{\varepsilon }_{0}\left({\varepsilon }_{d} +\frac{{\varepsilon }_{I}{l}_{f}}{{l}_{I}}\right)}.$$

For *θ* ≠ 0 and *θ*_*I*_ ≠ 0, Eq. (2) is *E*_*d*_ = − *P*_S_ cos *θ*/*ε*_0_(*ε*_*d*_ + *ε*_*I*_*l*_*f*_/*l*_*I*_).

Equation (2) yields also *E*_*d*_ in FE capacitors, because equations of continuity of electric flux similar to the above hold; A short-circuited FE capacitor is modelled as a perfect-metal/insulator(*l*_*I*_/2)/FE/insulator(*l*_*I*_/2)/perfect-metal^29^, where the perfect metal refers to a metal with zero screening length and the thickness of each screening layer *λ* is *l*_*I*_/2. Assuming *P*_I_ = *ε*_*I*_*ε*_0_*E*_*I*_ in screening layer, Eq. (2) is applicable and yields the Mehta formula^4^ with *ε*_*d*_ = *ε*_*f*_ and *l*_*I*_ = 2*λ*. For FE capacitors with *θ* ≠ 0, the formula beneath Eq. (2) is applicable. Because we neglected the electronic interactions at the metal/FE interface of 1–2 unit-cell, e.g. quantum mechanical smearing^30^, the formula for capacitors may be inaccurate for *l*_*f*_ < several unit-cells.

The nominal FE thickness *l*_*f*_ is the distance between the center position of a top ion and that of a bottom ion, but twice of the atom radius ~ 0.5 Å × 2 should be added. In case of FE/vacuum, this correction was examined by considering the smear-out of electrons into vacuum^29^; When *λ*_smear_ (~ 0.8 Å) is the distance between an outermost electron density and a center of ion position (“Methods”), FE thickness appropriate for electrostatics is *l*_*f*_^*eff*^ = *l*_*f*_ + 2*λ*_smear_. As seen below, Eqs. (1) and (2) can be valid down to a few nm *l*_*f*_ in case of FE/vacuum. Additionally, surface buckling layer is electrostatically a dipole layer, and its net charge is zero. Therefore, even in the presence of buckling layer, these formulas for 1D are also valid, by regarding buckling layer as dead layer (“Methods”); The effective thickness is *l*_*f*_^*eff*^ − 2*l*_*buckle*_, where *l*_*buckle*_ is the thickness of a buckling layer ~ 1–2 unit-cells.

## Results

### Estimation of *ε*_*d*_

*ε*_*d*_ was examined through the comparison of ab initio* E*_*d*_ with *E*_*d*_ of Eq. (1) or (2) that uses different values of *ε*_*d*_. For FE/vacuum (*P*_*I*_ = 0), *P*_S_ in Eq. (1) was the rigorously calculated *P*_S_ of the slab by Berry phase. For FE/insulators and FE capacitors, *P*_S_ and *P*_*I*_ in Eq. (2) were calculated ab initio. Therefore, all the parameters in Eqs. (1) and (2) except for *ε*_*d*_ are accurately known.

**Figure 3:**
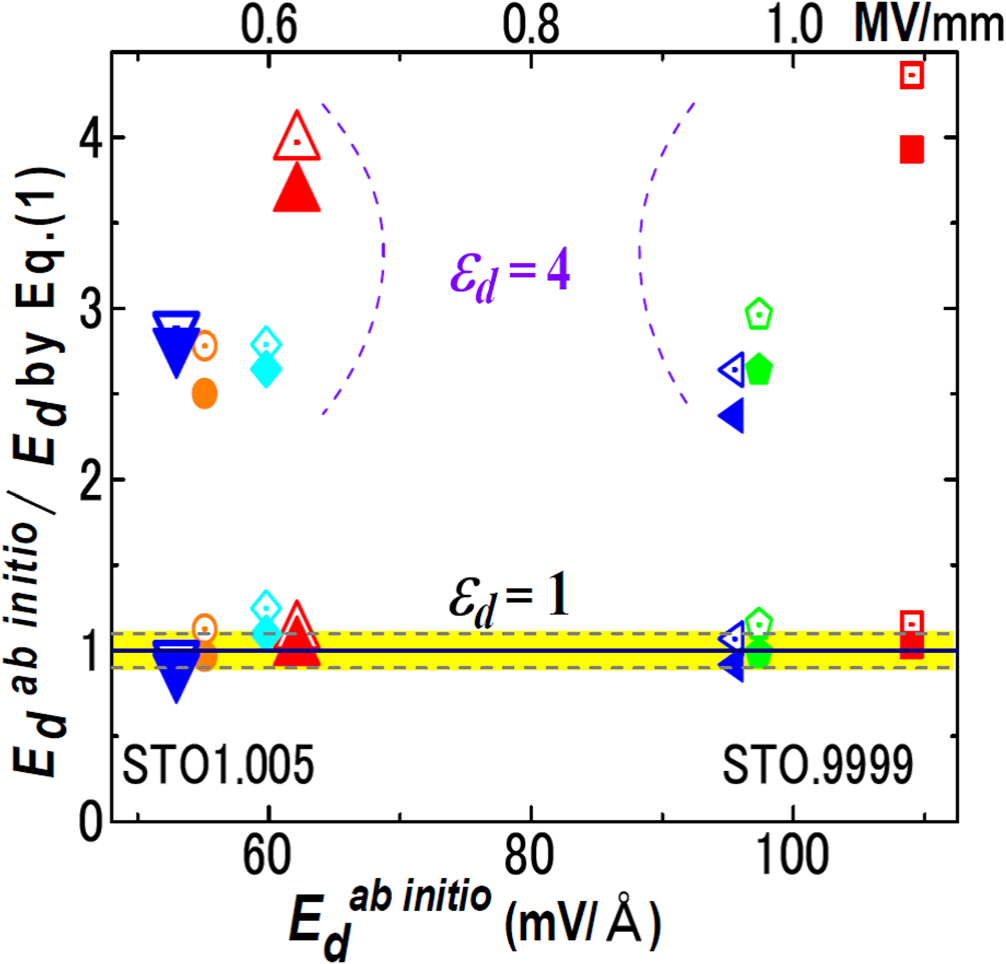
FE/vacuum. Comparison of *E*_*d*_^*ab initio*^ with Eq. (1) with *ε*_*d*_ = 1, 4 for STO1.005/vacuum and STO0.9999/vacuum. The yellow band shows the range of 1 ± 0.1. The filled and open symbols represent data in which *l*_*f*_ in Eq. (2) is estimated with *λ*_smear_ = 0.8 Å and *λ*_smear_ = *c*/2, respectively. The shape of symbol indicates a slab structure: For STO1.005, orange circles, red triangles, light blue diamonds, and blue inverted triangles correspond to 7-unit-cell-STO with *l*_V_ = 30 Å, 7-unit-cell-STO with *l*_V_ = 200 Å, 10-unit-cell-STO with *l*_V_ = 30 Å, and 10-unit-cell-STO with *l*_V_ = 100 Å, respectively. For STO0.9999, red squares, green pentagons, and blue 90°-rotated triangles correspond to 5-unit-cell-STO with *l*_V_ = 300 Å, 6-unit-cell-STO with *l*_V_ = 30 Å, and 7-unit-cell-STO with *l*_V_ = 30 Å, respectively.

Figure 3 compares *E*_*d*_’s by Eq. (1) with *E*_*d*_^*ab initio*^’s, where Eq. (1) uses *ε*_*d*_ = 1, 4. Here, *ε*_*d*_ = 4 is the lower bound of electronic permittivity of STO^29^. *E*_*d*_^*ab initio*^ is accurate for a long *l*_*f*_, and data for *l*_*V*_ ≫ *l*_*f*_ reflects the effect of *ε*_*d*_ explicitly because of *E*_*d*_ ~ − *P*_S_/*ε*_0_*ε*_*d*_. Large symbols in Fig. 3 show the data points satisfying both conditions and, hence, are important.

In Fig. 3, Eq. (1) with *ε*_*d*_ = 1 agrees with *E*_*d*_^*ab initio*^ within 10% always for *λ*_smear_ = 0.8 Å and mostly for *λ*_smear_ = *c*/2. The difference between *E*_*d*_’s for *λ*_smear_ = 0.8 Å and = *c*/2 provides typical error bar and is approximately 10%. Equation (1) with *ε*_*d*_ = 4 deviates from *E*_*d*_^*ab initio*^’s by more than 140%, and the deviations increase monotonically with *ε*_*d*_. Additionally, Eq. (1) yields the potential difference *ϕ*_max_ =|*E*_*d*_|*l*_*f*_ = *P*_S_/*ε*_0_(*ε*_*d*_/*l*_*f*_ + 1/*l*_*V*_), which, with *ε*_*d*_ = 1, quantitatively agrees with bandgap *Eg* that decreases with *l*_*f*_ and *l*_*V*_ in Fig. 1e.

**Figure 4:**
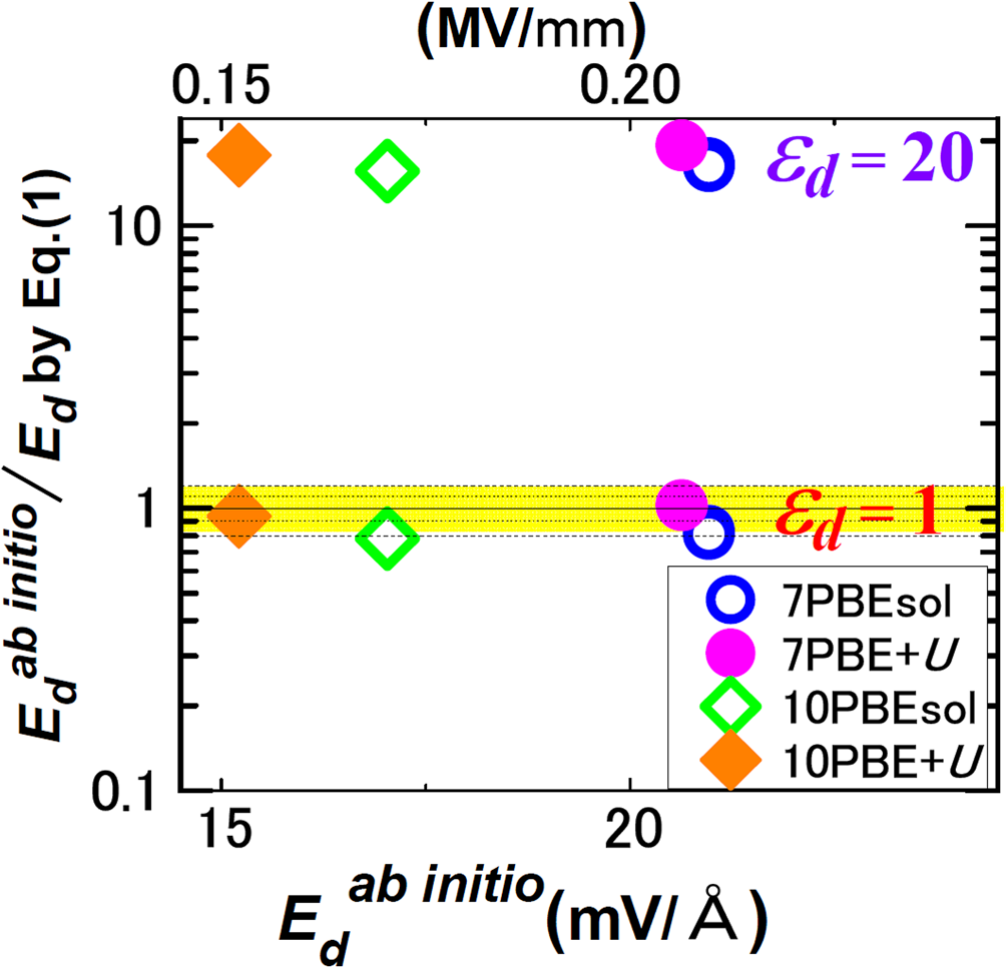
FE/paraelectric. Comparison of *E*_*d*_^*ab initio*^ with Eq. (1) with *ε*_*d*_ = 1, 20 for BTO/5-unit-cell-STO. The yellow band shows the range of 1 ± 0.2, and the dotted lines inside of the band show the range of 1 ± 0.1. Inset explains the BTO thickness *l*_*f*_ in unit-cells and ab initio method (PBEsol or PBE + *U*).

For BTO/STO, Fig. 4 compares *E*_*d*_’s by Eq. (1) with *E*_*d*_^*ab initio*^’s, where Eq. (1) uses ab initio* P*_S_ and ab initio* P*_*I*_. For *ε*_*d*_ = 1, Eq. (1) agrees within ± 20% with *E*_*d*_^*ab initio*^. In particular, the agreements are within ± 6% in the results by PBE + *U* (a method of ab initio calculation (“Methods”)). Equation (2) with *ε*_*d*_ = 20 deviated by more than 1000% from *E*_*d*_^*ab initio*^. The deviation increased monotonically with *ε*_*d*_, whereas *ε*_*f*_ > 20 is usual for inorganic FEs^4,5,7–10,16,17^.

**Figure 5:**
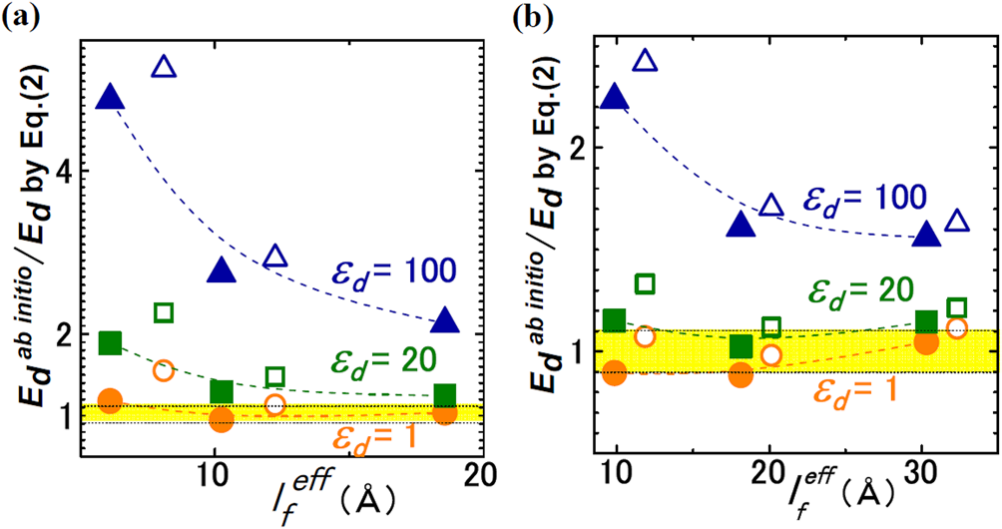
FE capacitor. Comparison of *E*_*d*_^*ab initio*^ with Eq. (2) with *ε*_*d*_ = 1 (orange), 20 (green), and 100 (blue) for (**a**) BTO/SrRuO_3_ and (**b**) BTO/Pt. The filled and open symbols represent the data for *l*_*f*_^*eff*^ = *l*_T-B_ − uc_BTO_, *l*_T-B_ − 1.5uc_BTO_, respectively.

For capacitors, Fig. 5a,b compare *E*_*d*_ by Eq. (2) with *E*_*d*_^*ab initio*^, where Eq. (2) uses ab initio* P*_S_ and *l*_*I*_/2*ε*_*I*_ ≈ 0.1 Å, 0.05 Å; *E*_*d*_’s by Eq. (2) with *ε*_*d*_ = 1 agrees best with *E*_*d*_^*ab initio*^’s. The disagreements of Eq. (2) with *E*_*d*_^*ab initio*^’s increases with *ε*_*d*_. The differences between open and filled symbols provide typical error bar and are 10–20%. Consequently, all the studied cases indicate *ε*_*d*_ = 1 (Figs. 3, 4, 5).

### Bridge between ***ε***_***d***_ = 1 and ***ε***_***f***_^13^

We showed *ε*_*d*_ = 1 and will use it below. By noting that electrical measurements of *P*_S_ are based on the change of charge per area in electrodes Δ*Q* induced by *E*_*ext*_, a bridge between *ε*_*d*_ = 1 and *ε*_*d*_ = *ε*_*f*_ will be shown. Because most studies^4–10,16,17^ are for FE capacitors, the followings are for 1D FE capacitors with *θ* = 0, which can represent FE/vacuum for *ε*_*I*_ = 1 and freestanding FE for *l*_*I*_/*ε*_*I*_ ≫ *l*_*f*_.

Using the total electric field in FE *E*_*f*_ and the total polarization of FE *P*, *P*_S_ ≡ *P*(*E*_*f*_(*E*_*ext*_ = 0)) and *E*_*d*_ ≡ *E*_*f*_(*E*_*ext*_ = 0). When the potential difference between the electrodes is *V*, Eq. (2) changes to *E*_*f*_(*V*) = *V*/(*l*_*f*_ + *l*_*I*_/*ε*_*I*_) − *P*(*E*_*f*_(*E*_*ext*_))/*ε*_0_(1 + *ε*_*I*_*l*_*f*_/*l*_*I*_)^13^. *E*_*I*_, the field in the screening layer of the electrode, is *E*_*I*_(*V*) = *V*/*ε*_*I*_(*l*_*f*_ + *l*_*I*_/*ε*_*I*_) + *P*(*E*_*f*_(*E*_*ext*_))*l*_*f*_/*ε*_0_*l*_*I*_(1 + *ε*_*I*_*l*_*f*_/*l*_*I*_).

*E*_*f*_(*V*) can be written as *E*_*f*_(*E*_*ext*_) = *E*_*ext*_ + *E*_*d*_(*E*_*f*_(*E*_*ext*_)), where *E*_*ext*_ ≡ *V*/(*l*_*f*_ + *l*_*I*_/*ε*_*I*_) (= *V*/*l*_*f*_ for *l*_*f*_ ≫ *l*_*I*_/*ε*_*I*_) and *E*_*d*_(*E*_*f*_(*E*_*ext*_)) ≡ − *P*(*E*_*f*_(*E*_*ext*_))/*ε*_0_(1 + *ε*_*I*_*l*_*f*_/*l*_*I*_) similar to Eq. (2).

*ε*_*f*_ is defined by *ε*_*f*_ − 1 = {*P*(*E*_*f*_(*E*_*ext*_)) − *P*_S_}/*ε*_0_(*E*_*f*_(*E*_*ext*_) − *E*_*d*_), where *E*_*d*_ = *E*_*f*_(*E*_*ext*_ = 0) = − *P*_S_/*ε*_0_ for *l*_*f*_ ≫ *l*_*I*_/*ε*_*I*_, and *ε*_*f*_ (≫ 1) is linear for |*E*_*ext*_| ≪ |*P*_S_|/*ε*_0_. Hence, *P*(*E*_*f*_(*E*_*ext*_)) = *P*_S_ + (*ε*_*f*_ − 1)(*ε*_0_*E*_*f*_(*E*_*ext*_) + *P*_S_) for *l*_*f*_ ≫ *l*_*I*_/*ε*_*I*_*.*

The substitution of this *P*(*E*_*f*_(*E*_*ext*_)) in the expression of *E*_*f*_(*E*_*ext*_) yields *E*_*f*_(*E*_*ext*_) = *E*_*ext*_/*ε*_*f*_ − *P*_S_/*ε*_0_ for *l*_*I*_/*ε*_*I*_ ≫ *l*_*f*_ (freestanding), suggesting that the measured permittivity is *ε*_*f*_*.*

We show an example: Δ*Q* = *ε*_*I*_*ε*_0_(*E*_*I*_(*V*) − *E*_*I*_(*V* = 0)) = *V*/(*l*_*f*_/*ε*_*f*_*ε*_0_ + *l*_*I*_/*ε*_*I*_*ε*_0_)^13^ from the above expression of *E*_*I*_(*V*). This is equal to Δ*Q* = *CV*, where *C* = (*C*_*f*_^−1^ + *C*_*I*_^−1^)^−1^ (*C*_*f*_ ≡ *ε*_*f*_*ε*_0_/*l*_*f*_, *C*_*I*_ ≡ *ε*_*I*_*ε*_0_/*l*_*I*_) is a series capacitance per area. In particular, for *l*_*f*_ ≫ *ε*_*f*_*l*_*I*_/*ε*_*I*_, Δ*Q* = *ε*_*f*_*ε*_0_*V*/*l*_*f*_ = *C*_*f*_* V*. Therefore, the permittivity of FE under *E*_*d*_ = *E*_*f*_(*E*_*ext*_ = 0) = − *P*_S_/*ε*_0_ is *ε*_*f*_.

Additionally, Eq. (2) with *ε*_*d*_ = 1 shows *D* = *P*_S_ − *P*_S_/(1 + *ε*_*I*_*l*_*f*_/*l*_*I*_), while *D* = *Q*. Because *l*_*I*_/*ε*_*I*_ ~ 0.1 Å, the difference between the real *P*_S_ and the measured *P*_S_ is detectable only for *l*_*f*_ < 10 Å. As for potential difference, Eq. (2) is well approximated by *E*_*d*_ = − *P*_S_*l*_*I*_/(*ε*_*I*_*ε*_0_*l*_*f*_) for *l*_*f*_ > 10 Å, because *l*_*I*_/*ε*_*I*_ is short ~ 0.1 Å. Therefore, the potential difference across the capacitor is independent of the FE thickness *l*_*f*_, when the quality of FE is independent of *l*_*f*_ and FE is ideally stoichiometric.

## Discussions

Here, we discuss only monodomain FE (Fig. 2c).

### Permittivity for non-polarization field (built-in field)

Because the polarization *P* in standard GLD theories^9,31–34^ are formulated with a single *total polarization*, *ε*_*d*_ = 1 should be used in standard GLD theories.

The preceding results have shown that the permittivity that expresses the change of *P* in response to *E*_*ext*_ is *ε*_*f*_ (≫ 1) even for FE under *E*_*d*_. By the same logic, the change of *P* by built-in internal field *E*^*bi*^ is also expressed by *ε*_*f*_ (≫ 1), where *E*^*bi*^ is not due to *P* or a dipole that is not expressed by *P*. *E*^*bi*^ exists in FEs by various mechanisms such as the diffusion potentials at *pn* and Schottky junctions and chemical orders, e.g. LaAlO_3_ in the polar catastrophe model.

For example, *P*_S_ = 0 and *E*_*d*_ = 0 in a bulk cubic BTO. However, *E*^*bi*^ ≠ 0, when the surfaces of a cubic BTO slab are asymmetrically terminated to form a dipole, e.g., BaO/TiO_2_/BaO/…/TiO_2_/BaO/TiO_2_. Hence, to achieve *E*^*bi*^ = 0, the present study used chemically symmetric slabs (Fig. 1a,b,g), e.g. BaO/TiO_2_/BaO/…/TiO_2_/BaO.

### Insulativity condition

For 1D-FE to remain insulating without artifactual screening, *el*_*f*_|*E*_*d*_| < *Eg* (*e*: elementary charge), for which Eq. (2) and *ε*_*d*_ = 1 yield 1/*l*_*f*_ > *eP*_S_ cos *θ*/*ε*_0_*Eg* − *ε*_*I*_/*l*_*I*_. Therefore, the condition of insulativity is one of the followings

3 $${l}_{I} \leq 1.8{\mathrm{\AA   }}{\varepsilon }_{I}\frac{{Eg}^{*}}{ {P}_\text{S}^{*}\left({T}_\text{C}\right){\mathrm{cos}}\,\theta },$$

4 $${l}_{f}<1.8{\mathrm{\AA }}{\left(\frac{{P}_\text{S}^{*}\left({T}_\text{C}\right){\mathrm{cos}}\,\theta }{{Eg}^{*}}-1.8{\mathrm{\AA }}\frac{{\varepsilon }_{I}}{{l}_{I}}\right)}^{-1},$$

where Eg* and *P*_S_*(*T*_C_) are bulk *Eg* normalized by 2 eV and *P*_S_ of bulk FE at *T*_C_ normalized by 10 μC/cm^2^, respectively, and the unit of *l*_*f*_ and *l*_*V*_ is Å. *P*_S_ of bulk FE at *T*_C_ approximates the critical *P*_S_ of FE that is about to become paraelectric by *E*_*d*_^12^. Equations (3) and (4) explain the insulativity of FEs in Fig. 1e,f.

### Freestanding insulating FE and HH–TT

The giant permittivity and large piezoelectric coefficients of FE are regarded as an *electrical softness* due to the tailing-effect of structural instabilities, i.e. phase transitions. This suggests that these properties are incompatible with an extremely stable FEs. For freestanding FEs (*l*_*I*_ = ∞, *θ* = 0), Eq. (3) yields *l*_*f*_ < 1.8 Å(*Eg*/2 eV)*/*(*P*_S_(*T*_C_)/10 μC/cm^2^). This suggests that freestanding FEs with normal bulk properties are FEs with metallic layer or insulating paraelectrics^12,15^ as explained by the following GLD analysis. This conclusion is valid also for HH–TT domains with *θ* = 0.

Standard GLD theories are based on a single polarization vector *P* as the order parameter. We approximate the polarization possibly missed in such GLD theory^29^ by an extra permittivity *ε*_*NG*_ − 1^35,36^, while *ε*_*NG*_ is speculatively close to electronic permittivity^29^. The GLD energy *F* of an *insulating* FE is *F* = (*T* − *T*_0_)*P*^2^/2C*ε*_0_ + *βP*^4^/4 + *γP*^6^/4 − *PE*_*d*_/2, where *T*_0_, *C*, *β*, *γ*, and *θ* are Curie–Weiss temperature, Curie constant, and GLD coefficients, respectively. The effect of strain can be incorporated in *T*_0_ and *β*^31–34,37^. Curie temperature *T*_C_ is *T*_0_ + Δ*T*, where Δ*T* = 3*β*^2^/16*γCε*_0_. For 2nd order transition, *γ* = 0, *β* > 0, and *T*_C_ = *T*_0_. The effect of *E*_*d*_ = − *P*_S_/*ε*_0_*ε*_*NG*_ is the change of *T*_0_ to *T*_0_ − *C*/*ε*_*NG*_ in *F*, where Eq. (2) with *ε*_*d*_ = *ε*_*NG*_ and *l*_*I*_ = ∞ is used.

Hence, the existence of freestanding FE that undergoes 2nd order FE transition is *T*_C_ > *C*/*ε*_*NG*_. This means *χ*_*GL*_ < *ε*_*NG*_/2 at *T* = 0, because *χ*_*GL*_ = *C/*2*T*_C_ at *T* = 0. The total permittivity *ε*_*f*_ = *χ*_*GL*_ + *ε*_*NG*_ at *T* = 0 is < 3*ε*_*NG*_/2 and < 7.5 for *ε*_*NG*_ = 5^29^.

Similarly, for FE undergoing 1st order FE transition, it is known that *χ*_*GL*_ = 1*/*{(*T* − *T*_0_)/*Cε*_0_ + 3*βP*^2^ + 5*γP*^4^}*ε*_0_ at *T* < *T*_C_. Stable state satisfies ∂*F*/∂*P* = (*T* − *T*_0_)/*Cε*_0_ + *βP*^2^ + *γP*^4^ = 0. Therefore, *χ*_*GL*_ = 1*/*(4*T*_0_/*Cε*_0_ − 2*βP*^2^)*ε*_0_ < *C/*4*T*_0_ at *T* = 0 (We assume *T*_0_ > 0), and *T*_C_ = *T*_0_ + Δ*T* = *T*_0_ + 3*β*^2^/16*γCε*_0_. Because Δ*T* ≪ *T*_0_ in almost all FEs^2, 31–34^, we may assume *T*_C_ < 2*T*_0_. Under this assumption, *χ*_*GL*_ < *C/*2*T*_C_ at *T* = 0, and *C* < *ε*_*NG*_*T*_C_ means *χ*_*GL*_ < *ε*_*NG*_/2 at *T* = 0. *ε*_*f*_ = *χ*_*GL*_ + *ε*_*NG*_ < 3*ε*_*NG*_/2 at *T* = 0, which is < 7.5 for *ε*_*NG*_ = 5.

These *ε*_*f*_’s of FEs undergoing 2nd and 1st transitions appear too small for experimentally observed bulk metal oxide FEs. Therefore, freestanding insulating FEs satisfying *C* < *ε*_*NG*_*T*_C_ are unlikely to exist, unless *ε*_*NG*_ is far larger than 5; That is, for freestanding FE materials, there exists virtually one choice between a partial loss of insulativity and a loss of FE.

### Design of freestanding insulating FE

For freestanding insulating FE (*θ* = 0), *C* < *ε*_*NG*_*T*_C_ was shown, while *Δϕ* ~ *P*_S_(*E*_*d*_)*l*_*f*_/*ε*_0_. Therefore, FE materials having a very large *ε*_*NG*_ (≫ 5) can retain FE and remain insulating, when ultrathin. Such FE materials are unlikely to exist. Alternatively, we may consider *electrically freestanding* FE or FE with clean surface that is not mechanically freestanding. In this case, *T*_0_ (~ *T*_C_) of common FEs increases to *T*_0_^*eff*^ by inplane strain, while ab initio calculations shows that *T*_0_^*eff*^ is much larger than those of standard GLD theories^37^. Therefore, heavily strained FE materials may retain FE and remain insulating, when ultrathin (Formula estimating an effective *T*_0_ from ab initio* P*_S_ is Ref. [88] of Ref.^37^). The above indicates that *ε*_*f*_ of such FE is extremely low for *E*_*d*_ = 0 but can be large for *E*_*d*_ ≠ 0, because the coefficient of the first term GLD energy *F* is (*T* − *T*_0_^*eff*^ + *C*/*ε*_*NG*_)/2C*ε*_0_ (freestanding).

### LiBeSb

**Figure 6:**
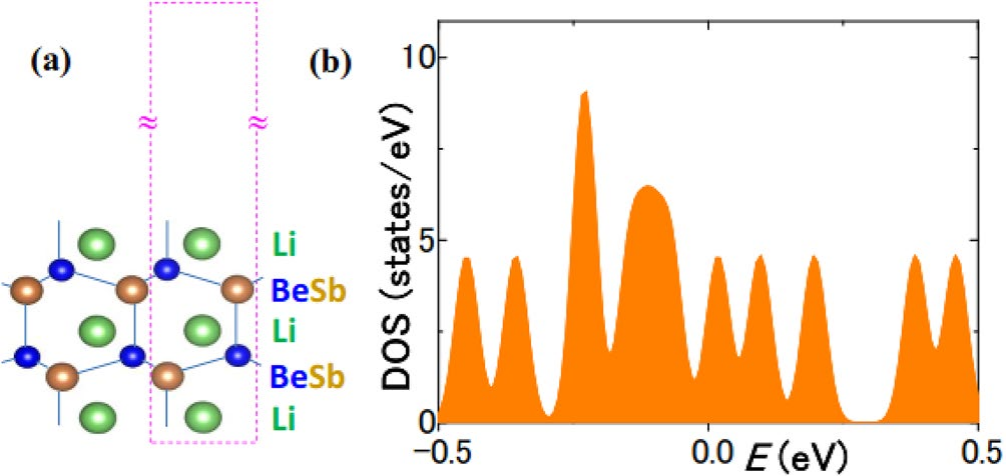
(**a**) Atomic model of LiBeSb/vacuum. (**b**) DOS showing metallicity of LiBeSb/vacuum.

LiBeSb with P6_3_mc symmetry^38^ is reported as a hyper-FE that retains both FE and insulativity in FE/paraelectric superlattices, which may contradict the above conclusion on insulativity and FE stability. Therefore, we ab initio calculated one-unit-cell LiBeSb (*l*_*f*_ = 6.08 Å) in vacuum (Fig. 6a). Figure 6b shows a metallic DOS of LiBeSb for *l*_*V*_ = 31.7 Å, while metallicity increases with *l*_*V*_. This is consistent with the above conclusion and the previous reports^14,15,39–41^. Equations (3) and (4) explain the insulativity of LiBeSb^38^ as the effect of adjacent dielectric. Actually, similar to LiBeSb, BTO/STO superlattices are insulating as in Fig. 1f, while BTO/vacuum is partially metallic^18^.

### Freestanding and free-surface FE: hidden mechanism

Mechanically freestanding FE is customarily referred to as freestanding; Ji et al.^42^ reported exceptionally intriguing results of the freestanding insulating BiFeO_3_ (BFO) that retains FE down to monolayer. This appears to contradict both the reports of metallicity at HH–TT domains of BFO and the present results, esp. the single choice between insulating paraelectric and partially metallic FE.

If *ε*_*d*_ = *ε*_*f*_ = 100, the potential difference *Δϕ* across freestanding insulating BFO of 1–4 unit-cell thickness with a moderate *P*_S_ ~ 20 μC/cm^2^ is 0.09–0.36 V by Eq. (2) with *l*_I_ = ∞, which allows this BFO to be insulating in agreement with Ji et al.^42^. For *ε*_*d*_ = 1, *Δϕ* increases by 100 times, by which BFO’s have to be partially metallic.

Hence, we shall look at the measurements of Ji et al.^42^. For freestanding FE, it was shown that the surface or boundary of 1 ~ 2 unit-cell thickness was metal and the rest was insulating FE^14,15,39,40^. So, the metallicity is detectable only by inplane conductance, which is absent in Ji et al.^42^. Second, because the crystallographic properties of FE with these metal layers was shown to be those of FE^14,15,39,40^, the crystallographic measurements of Ji et al.^42^ do not exclude metallic layers. Third, because piezoelectric measurements use bottom and top electrode (or tip) and may move ions^43^, those by Ji et al.^42^ are not that of freestanding FE. Consequently, all the measurements of Ji et al.^42^ do not contradict the conclusion of the present paper.

More importantly, the interdisciplinarity of nano FE hides true mechanisms. In the present case, “freestanding” is defined by surface science and electrostatics. For example, Fong et al. found monodomain FE of 3 unit-cell thickness as opposed to *E*_*d*_*-*limited domain and size effect, which was later attributed to adsorbates^44^. This agrees with recent ab initio study^45^. Further, photoemission spectroscopy in UHV showed that SrTiO_3_ surface was covered by adsorbates even in ultrahigh vacuum (UHV)^46^. Actually, the free surface with *P*_S_ ⊥ surface is insulator-like in air and metallic in UHV when cleaned^14^. Because the insulating freestanding FE^42^ was exposed to air and water, we suggest adsorbates as its hidden mechanism.

## Conclusion

We studied the electrostatics of *E*_*d*_, especially, the value of permittivity *ε*_*d*_ in the formula of *E*_*d*_ by ab initio simulations, where ab initio* P*_S_ corresponded accurately to experimental *P*_S_. For this, the standard theoretical assumptions: pure, insulating, stoichiometric, and clean FEs were used. To validate the analyses of *E*_*d*_ based on electrostatics, we concentrated on the formulas of *E*_*d*_ for accurate ab initio total *P*(*E*_*f*_(*E*_*ext*_ = 0)) that contained various atomic effects and corresponded to experimental *P*_S_. Further, we focused on the simplest cases of *E*_*d*_: freestanding 1D-FE, HH–TT domains, and superlattices that mimicked inhomogeneous FE and FE/dielectric.

The present ab initio simulations showed *ε*_*d*_ = 1 ± 0.06–1 ± 0.2. That is, *ε*_*d*_ = 1 should be applied to experimental and standard-GLD *P*_S_’s. A contradiction between *ε*_*d*_ = 1 and *ε*_*d*_ = *ε*_*f*_ was resolved by a bridge; Even under *E*_*d*_, the permittivity for *E*_*ext*_ and built-in field *E*^*bi*^ was *ε*_*f*_. Therefore, if a study requires *ε*_*d*_ ≫ 1^4–10,16,17^, the value of *P*_S_ is incorrect, the values of the parameters are inappropriate, or, most likely, hidden screening mechanisms exist^14,15,43–48^.

For freestanding insulating FEs (*l*_*I*_ = ∞), Eq. (2) yields *E*_*d*_ = − *P*_S_/*ε*_0_ (or *E*_*d*_ = − *P*_S_cos *θ*/*ε*_0_), while, for HH–TT insulating domains, Eq. (1) with *P*_*I*_ = − *P*_S_ and *l*_*I*_ = *l*_*f*_ yields the same *E*_*d*_. Therefore, when the effects at surface of 1–2 unit-cell is unimportant, freestanding FEs are electrostatically exactly identical with HH–TT domains.

Consequently, both the electrostatic energy of *E*_*d*_ and the FE free energy of insulating freestanding and HH–TT FEs scale linearly with *l*_*f*_. This implies that the stability of 1D-freestanding and HH–TT *insulating* FEs is independent of size^12,15^, when the energy increase by surface effect and domain walls energy is ignored. A strain effect to overcome this restriction was suggested.

**Figure 7:**
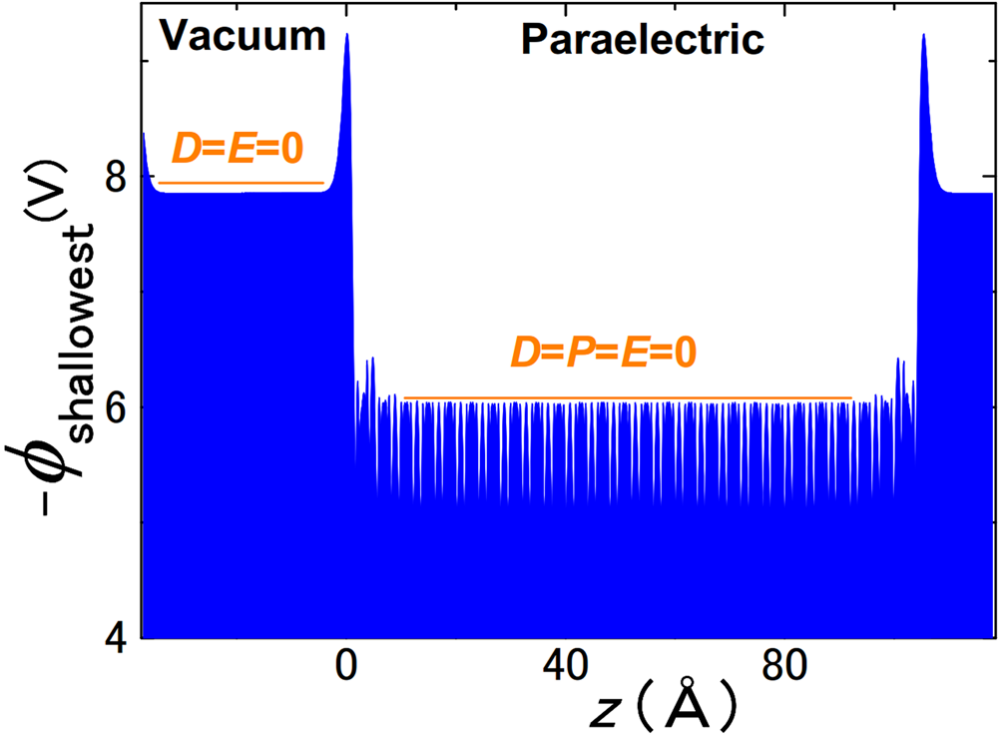
Ab initio electrostatic potential *ϕ* of paraelectric insulating BTO in vacuum showing peaks due to surface dipoles formed by electron smear-out and buckling. In this calculation of 26.5 unit-cell-BTO slab, all the ion positions are fully relaxed, and the insulting paraelectric state is the lowest energy state. *ϕ* = 0 in FE means *E*_*d*_ = 0, for which Eq. (2) suggests *P*_S_ = 0. *P*_S_ = 0 is also directly confirmed by ab initio calculations. Similarly, *E*_*d*_ = 0 in vacuum. Therefore, *D* = 0 in FE and vacuum.

Because *l*_*f*_*P*_S_/*ε*_0_ < *E*g/*e* by *ε*_*d*_ = 1, the insulativity required an extremely small bulk *P*_S_ ≪ 1 μC/cm^2^ or paraelectricity (Fig. 7). Alternatively, the stability of 1D-freestanding and HH–TT FEs for of (*θ* ≈ 0) required a partially metallic FE. This conclusion verified the previous results^14–16, 39–41^ in a material-independent manner and was confirmed also for hyper-FE LiBeSb that was reported to be insulating in FE/paraelectric^38^. This conclusion appeared inconsistent with “freestanding” monolayer BFO^42^. But, the examinations of experimental procedures^42^ suggested adsorbates as a hidden mechanism^43–46^.

The electrostatic formulas of *E*_*d*_ (Eqs. 1 and 2) were valid down to a several unit-cell scale (Figs. 3, 4, 5), when atomic-scale surface effects, e.g. interactions with electrodes^11,28^ were unimportant. Even with buckling at FE surfaces, these formulas can be valid by regarding buckling layers as dead layer.

## Methods

### Ab initio calculations

In the examinations of FE/vacuum, the results of SrO-terminated STO slabs are presented, because they have *Eg* wider than *Eg* of the TiO_2_-terminated STO slabs (Fig. 1e). To enforce FE, the ion-positions in the STO/vacuum slabs were not optimized, because, otherwise, FE disappears (Fig. 7). Therefore, the STO unit-cells in the slabs retained the ion positions of bulk STO1.005 or STO.9999. These calculations of STO/vacuum were only for the examination of *E*_*d*_ and *ε*_*d*_ and do not correspond to standard experiments. *P*_S_ of STO1.005 in the slab was typically 1 μC/cm^2^.

The models of FE/paraelectric are BTO/STO superlattices. All the calculated forces were < 1 meV/Å after geometry relaxation, and the *a*-axis lattice constant of STO was expanded by 1.1–1.3%. The bulk STOs that had these *a*-axis lattice constants were paraelectric^19^. The *a*-lattice constant of BTO/SrRuO_3_ and BTO/Pt capacitor was fixed at the theoretical *a*-axis lattice constant of cubic STO and bulk tetragonal BTO, respectively, and all other ion positions were relaxed (Fig. 1g). The atomic models of BTO/SrRuO_3_ are similar to BTO/STO (Fig. 1b). The use of the theoretical *a-*lattice constant of cubic STO corresponds to the thin films on STO substrates. The surfaces of the BTO and SrRuO_3_ were TiO_2_ and SrO, respectively, and Pt atoms at the interface aligned with O atoms of TiO_2_ plane.

The present study is about the formulas of *E*_*d*_ for given structure parameters. Here, the change of *P*_S_ by the interactions in the slabs is included consistently in these formulas by the use of ab initio* P*_S_ in these formulas.

The ab initio calculations with VASP^20–22^ used the projector augmented wave method^23^ with a Monkhorst–Pack^24^ mesh of 8 × 8 × 2 for slabs and an energy cutoff of 650 eV. PBEsol functional^25^ was used, unless otherwise mentioned. Ab initio* P*_S_ was calculated through Berry phase^26^. The results of BTO/STO were reexamined with PBE functional^27^ with Hubbard *U* (PBE + *U*)^28^, which was used also for BTO/Pt. In the slab calculations, graphic processing units acceleration^49,50^ was used. The supercells were produced by VESTA^51^.

### Accurate estimation of ***P***_S_ under ***E***_***d***_

For correct *ε*_*d*_, accurate estimations of a total polarization *P*_S_ under *E*_*d*_ are essential. Because we compare Eqs. (1) and (2) using ab initio* P*_S_ with *E*_*d*_ for the ion positions same as those of this *P*_S_, the accuracy of *P*_S_ for *given ion positions* matters*.*

Berry phase calculation of *P*_S_ *for given ion positions is accurate but only possible for insulators.* For example, the present Berry phase calculations yields *P*_S_ of bulk BaTiO_3_ that agree with experimental *P*_S_ within 4%, when experimental ion positions and lattice constants of at 303 K are used^37^.

Therefore, to obtain accurate *P*_S_*, the dipole moment of a whole slab was calculated with Berry phase*; We treated these slabs as unit-cells to apply Berry phase calculations directly, unlike conventional approaches. *P*_S_ was obtained by dividing the dipole moment by the volume of FE part of the slab. These *P*_S_’s were referred to “rigorously calculated *P*_S_’s of the slab” and obtained for all the FE/vacuum and BTO/STO slabs. Here, STO1.005 and STO.9999 slabs are insulating, allowing accurate Berry phase calculations.

Additionally, *P*_S_ of the unit-cell that possessed exactly the same ion positions as those in the slab was calculated with Berry phase and, then corrected with atomic polarization by *E*_*d*_ by the procedures in Ref.^29^. These *P*_S_’s agree perfectly with “rigorously calculated *P*_S_’s of the slab”, which further confirmed the accuracy of the present *P*_S_’s of FE/vacuum and BTO/STO. These corrected *P*_S_’s^29^ were used for capacitors. Therefore, in the present study, *P*_S_’s are accurate *total P*_S_’s and self-consistent with *E*_*d*_. Hence, *P*_S_’s in Figs. 3, 4 and 5 are accurate.

### Validity of continuity of electric flux and surface dipoles

Equations (1) and (2) are applicable to the regions much larger than unit-cell. Here, the peaks of 1.5 Å width at the surface in Fig. 1d may be suspected to invalidate Eqs. (1) and (2). These peaks are due to effective surface dipoles caused by electron tunneling smear-out; Surface electrons smear out in vacuum, making positive charge density inside the surface and negative charge density in vacuum.

The heights and shape of the two peaks from the baseline (yellow line in Fig. 1d) are the same (1.53 V). This means *σ*^+^_R_ = *σ*^+^_L_ and *σ*^−^_R_ = *σ*^−^_L_ as expected from their origin, where *σ*^+^_R_, *σ*^−^_R_, *σ*^+^_L_, and *σ*^−^_L_ are positive and negative charge densities that yield the right and left peak, respectively. Because of the charge neutrality of FE, *σ*^+^_R_ + *σ*^+^_L_ + *σ*^−^_R_ + *σ*^−^_L_ = 0, i.e. *σ*^−^_R_ + *σ*^+^_R_ = 0. Therefore, the continuity of the electric fluxes *D*_*FE*_ in FE and *D*_*I*_ in *I*_*adj*_ (Fig. 1b) is *D*_*FE*_ − *D*_*I*_ = *σ*^−^_R_ + *σ*^+^_R_ = 0, i.e. the continuity of electric flux *D*_*FE*_ = *D*_*I*_, where *D*_*FE*_ = *P*_S_cos *θ* + *ε*_*d*_*ε*_0_*E*_*d*_ and *D*_*I*_ = *P*_*I*_ cos *θ*_*I*_ + *ε*_0_*E*_*I*_. A clearer example is shown in Fig. 7, which evidently shows the continuity of electric flux and, hence, validates the use of Eqs. (1) and (2).

Because surface buckling in vacuum is electrostatically dipole due to ion displacements, the arguments exactly the same as the above hold. Therefore, the electric flux of the inside *D*_*FE*_ and the outside *D*_*V*_ of the buckling layer is continuous (*D*_*FE*_ = *D*_*V*_).

### Effective ***l***_***f***_ (***l***_***f***_^***eff***^)

For FE/vacuum, the effective *l*_*f*_ (*l*_*f*_^*eff*^) was estimated from the planer averaged electron density *ρ* profiles^29^. Below, *z* = 0 corresponds to the position of bottom ion. Because *ρ* at *z* = − 0.8 Å was same as the minimum *ρ* of inner part in all the *ρ*–*z* curves, the region of *z* = 0 ~ − 0.8 Å was considered as a part of FE (*λ*_smear_ = 0.8 Å), and *l*_*f*_^*eff*^ was *l*_*f*_^*eff*^ = *l*_*f*_ + 2*λ*_smear_. In addition, *λ*_smear_ = *c*/2 (half unit-cell) was also tested, and *l*_*V*_ = *l*_SC_ − *l*_*f*_^*eff*^. For BTO/STO, *l*_*f*_ was defined as the distance between the top and bottom Ti ions of BTO (Fig. 1b), and *l*_*I*_ = *l*_SC_ − *l*_*f*_.

For FE capacitors, *l*_*f*_^*eff*^ was *l*_T-B_ − uc_BTO_ (outermost ions are Ti, uc_BTO_: length of a BTO unit-cell), for which the quantum mechanical smearing^29^ may be responsible. The estimations with *l*_T-B_ − 1.5uc_BTO_ were also tested. The effective thicknesses of the screening layer, i.e. the effective passive layer *l*_*I*_/2*ε*_*I*_ of BTO/SrRuO_3_ and BTO/Pt were estimated as 0.1 Å and approximately 0.05 Å, respectively^29^.

## Data Availability

The data required to reproduce these findings can be provided upon reasonable requests to the corresponding author.
